# Calling for an exponential escalation scheme in vaccine development for COVID-19

**DOI:** 10.1007/s00228-020-02933-w

**Published:** 2020-06-16

**Authors:** Martin Wehling

**Affiliations:** Institute of Clinical Pharmacology Mannheim, Medical Faculty Mannheim, University of Heidelberg, Theodor-Kutzer-Ufer 1-3, 68167 Mannheim, Germany

**Keywords:** COVID-19, Vaccination, Seamless trial design, Adaptive design, Exponential growth

## Abstract

**Purpose:**

COVID-19 as a pandemic calls for rapid development of vaccines.

**Methods:**

Here a proposal of a seamless, adaptive, phase 1–3 trial for accelerated vaccine development is described.

**Results:**

Starting at 10, the number of vaccinated volunteers would exponentially increase by tenfold at an interval of 2 weeks; close surveillance of antibody responses, safety and efficacy is necessary. After only 16 weeks, general vaccination would be feasible if supply meets the demand.

**Conclusion:**

A COVID-19 vaccine would be rapidly available at a slightly increased risk for undetected late side effects or insufficient efficacy if compared with standard vaccine development schemes.

COVID-19 is a pandemic that may kill millions of people.

Obviously, the only true solution is a successful vaccine, but the timing of its realization is at least as crucial as its existence. A successful intervention has to be realized under an enormous developmental time pressure.

The biggest threat to the timely development of a vaccine is to adhere to common rules of clinical development, mainly characterized by subsequent phases (1–3) of clinical testing before marketing approval. This process may take 3–5 years.

Some accelerated testing schemes have been suggested in recent weeks [1], but as a clinical pharmacologist engaged in translational medicine and in the design of smart early clinical trials [2], I would like to propose an even more radical deviation from normal standards.

The pre-requisite for human trials of a vaccine is its successful testing in animals with three major goals:

1. The induction of desired antibodies known to be induced by the real infection
2. Absence of antibody-dependent enhancement meaning non-neutralizing antibodies that otherwise may enhance disease-related damage
3. Absence of structural homologies of antibody targets with protein structures normally present in humans, analogous to the still disputed induction of narcolepsia by the influenza vaccine Pandemrix due to a structural homology with the hypocretin receptor [3]

I suggest exponential exposure starting with 10 healthy volunteers, testing of antibody responses and safety after 14 days; if no stoppers [lack of adequate (see points 1–3 above) antibody response, intolerable side effects] occur, the number of vaccinated people will be increased by tenfold in each subsequent step, in this case to the next cohort of 100 people. Each transition may include a modification of dosing, reflecting the antibody response measured. Only 12 weeks after the first vaccination, one million people would have been vaccinated; at this point, side effects have been monitored in 10 people for 12, in 100 people for 10, in 1000 people for 8 and in 10,000 people for 6 weeks. As a rule of thumb, a rare side effect occurring within 6 weeks would have been detected at a likelihood of 1/10000 × 3 = 3000 cases. Thus, even rare (though not very rare or late) side effects would have been noticed, stopping further escalation. For safety reasons, this scheme should be restricted to inactivated vaccines as the risks for live-attenuated vaccines may be greater.

Vaccination efficacy must be monitored in parallel; ideally, the early stages of the study should be placed in countries with low risk of infection. At stage 3 + (1000 + people) when primary efficacy has been established at the antibody level and safety is no issue to this point, volunteers living in countries with high infection rates should be vaccinated. If spontaneous infection rates drop below average in vaccinated people, this would be an early sign for efficacy. Such efficacy results may be visible already at the end of the 10,000–100,000 people level, meaning after 8–10 weeks.

This seamless, adaptive phase 1–3 study design relies on continuous observation, and testing of all participants may be up the 1,000,000 people level. Obviously, at week 16 (4 months, 100 million people) or even earlier if results are compelling, pandemic (unlimited) vaccination could be performed. Thus, this vaccine would be available worldwide 16 weeks after the end of successful animal testing. Participating people would have to be informed that:

- This design is relatively insensitive to late side effects occurring beyond 8–12 weeks.
- The escalation may put people at risk before side effects or insufficient efficacy may become visible.

Obviously, many (120 +) vaccine projects have been started, and such escalation schemes could be performed competitively for those fulfilling the above criteria. Studying various vaccines in parallel to identify the best candidate is possible as the COVID pandemic is still rapidly expanding in several countries, in particular in Brazil, for example.

A recent proposal to infect healthy people even in the placebo group by COVID-19 [1] is truly heroic given the fact that even young people may die of the disease and would be unnecessary under the auspices of this proposal.

It has to be kept in mind that not only regional political interests but also difficulties in producing large quantities of vaccines may interfere with the presently most urgent goal of medical care around the planet: rapidly vaccinating a large share of the global population against COVID-19. Political discussions about vaccine distribution and provision of production capacities should be preemptively addressed as otherwise we may witness another ethical tragedy: a successful vaccine could have been developed, but will not be available for all countries, and shortage of supply may aggravate the disaster of politically driven restrictions of distribution.

We applaud COVID heroes, e.g. in the health care system. Vaccine development is not possible without the participation of consenting volunteers. If the extreme time pressure leads to accelerated clinical trials, volunteers may face a slightly increased risk (see above). We have to ask them if they would be willing to take this risk for ethical reasons, in that they may save the lives of many COVID victims. Thus, volunteers in vaccine studies should be applauded as well.
